# Cardiovascular disease management and healthcare delivery for people experiencing homelessness: a scoping review

**DOI:** 10.1186/s12913-024-11503-0

**Published:** 2024-09-17

**Authors:** Jillian Macklin, Bethel Samson, Alex Zsager, Heather Ross, Andrew Pinto, Jennifer L. Gibson

**Affiliations:** 1 Institute of Health Policy, Management, and Evaluation, Dalla Lana School of Public Health, Toronto, ON Canada; 2 Temerty Faculty of Medicine, University of Toronto, Toronto, ON Canada; 3 MAP Centre for Urban Health Solutions, Unity Health Toronto, Toronto, ON Canada; 4 Peter Munk Cardiac Centre, University Health Network, Toronto, ON Canada; 5 Department of Family and Community Medicine, University of Toronto, Toronto, ON Canada; 6 Division of Clinical Public Health, Dalla Lana School of Public Health, Toronto, ON Canada; 7 Joint Centre for Bioethics, University of Toronto, Toronto, ON Canada

**Keywords:** Homelessness, Cardiovascular disease, Chronic disease management, Vulnerable population

## Abstract

**Background:**

People experiencing homelessness have increased prevalence, morbidity, and mortality of cardiovascular disease (CVD), attributable to several traditional and non-traditional risk factors. While this burden is well-known, mainstream CVD management plans and healthcare delivery have not been developed with people experiencing homelessness in mind nor tailored to their unique context. The overall objective of this work was to explore and synthesize what is known about CVD management experiences, programs, interventions, and/or recommendations specifically for people experiencing homelessness.

**Methods:**

We conducted a scoping review to combine qualitative and quantitative studies in a single review using the Arksey and O’Malley framework and lived experience participation. We performed a comprehensive search of OVID Medline, Embase, PsychINFO, CINAHL, Web of Science, Social Sciences Index, Cochrane, and the grey literature with key search terms for *homelessness*, *cardiovascular disease*, and *programs*. All dates, geographic locations, and study designs were included. Articles were analyzed using conventional content analysis.

**Results:**

We included 37 articles in this review. Most of the work was done in the USA. We synthesized articles’ findings into 1) barriers/challenges faced by people experiencing homelessness and their providers with CVD management and care delivery (competing priorities, lifestyle challenges, medication adherence, access to care, and discrimination), 2) seven international programs/interventions that have been developed for people experiencing homelessness and CVD management with learnings, and 3) practical recommendations and possible solutions at the patient encounter level (relationships, appointment priorities, lifestyle, medication), clinic organization level (scheduling, location, equipment, and multi-disciplinary partnership), and systems level (root cause of homelessness, and cultural safety).

**Conclusions:**

There is no ‘one-size-fits all’ approach to CVD management for people experiencing homelessness, and it is met with complexity, diversity, and intersectionality based on various contexts. It is clear, however, we need to move to more practically-implemented, community-driven solutions with lived experience and community partnership at the core. Future work includes tackling the root cause of homelessness with affordable housing, exploring ways to bring cardiac specialist care to the community, and investigating the role of digital technology as an avenue for CVD management in the homeless community. We hope this review is valuable in providing knowledge gaps and future direction for health care providers, health services research teams, and community organizations.

**Supplementary Information:**

The online version contains supplementary material available at 10.1186/s12913-024-11503-0.

## Introduction

Cardiovascular disease (CVD) is the leading cause of mortality, accounting for nearly 31% of all deaths worldwide [1]. CVD is largely treatable or controllable if patients have appropriate access to medication, multi-disciplinary healthcare support, and self-management skills, and the life expectancy of people with CVD has improved due to advances in prevention, treatment, and management of the disease [2–5]. However, many disparities exist for the access and benefit of CVD care due to complex interactions between environmental, physical, psychosocial, and economic determinants. This is especially relevant to those defined as part of a group with structural vulnerability [6–8], and notably among people experiencing homelessness [9–11].

The United Nations estimate that 100 million people were homeless worldwide in 2005 [12]. The Canadian Alliance to End Homelessness defines homelessness “as the situation of an individual, family, or community without stable, safe, permanent, appropriate housing, or the immediate prospective means and ability of acquiring it” [13]. This includes people who are unsheltered (e.g., outside), emergency-sheltered (e.g., shelter/drop-in centre), provisionally accommodated (e.g., on a friend’s couch or in a car), or those at immediate risk of losing shelter (e.g., threatened eviction or precarious economic situation). These situations could be crisis, episodic, or chronic [13].

People experiencing homelessness have a three times higher CVD prevalence and a significantly increased CVD mortality rate (ranging from 2-6x) than the housed population [14]. These outcomes are attributable to several heightened traditional risk factors (e.g., smoking, uncontrolled comorbidities), non-traditional risk factors (e.g., chronic stress, IV drug use, severe mental illness), difficulty of CVD diagnosis (e.g., late presentation, lack of usual care access, stigmatization), and difficulty managing CVD (e.g., care fragmentation, medication adherence) [9]. The homeless population also intersects and has an over-representation of various other structurally vulnerable populations facing their own systemic racism and discrimination, including Indigenous groups, racialized groups, LGBTQ + groups, refugees, and recently incarcerated individuals [9, 14].

## Rationale

The burden of CVD on the homeless community is well-known. However, mainstream CVD management plans and healthcare delivery have not been developed with people experiencing homelessness in mind [9, 15]. Research has shown that people experiencing homelessness have better experiences and health outcomes when healthcare programs are tailored to their unique situational needs [16, 17]. It is likely that healthcare groups and community organizations have begun to incorporate lived experience perspectives into developing and piloting CVD care practices to help the local community, that are not yet captured in the literature. Therefore, we conducted a scoping review to synthesize the findings from these experiences and programs.

This work builds on a few recent publications. In 2018, Hanlon et al. reviewed interventions for homeless individuals with the intent of improving management of non-communicable diseases and communicable diseases requiring long-term care. Their work focused mainly on infectious diseases such as tuberculosis, HIV, and hepatitis, without any studies on CVD [17]. Also in 2018, Baggett et al. wrote a literature review on CVD and homelessness, including mortality rates, risk factor burden, barriers to diagnosis and management, clinical recommendations, and tailored models of care [9]. However, they did not systematically search the full literature, and did not include studies on lived experience or CVD-specific programs/interventions, aside from smoking cessation and diabetes. Finally in 2020, Al-Shakarchi et al. conducted a systematic review and meta-analysis of observational and interventional studies to identify the risk of CVD in homeless versus housed individuals, as well as interventions for CVD in homeless individuals. They did not find any specific CVD pathways or interventions for homeless individuals and concluded a significant knowledge gap and urgent attention needed for integrated CVD care for this population [14]. However, their search strategy did not include the grey literature (e.g., reports, community websites, etc.), and they excluded qualitative and descriptive studies. Given the limitations and different scopes of these previous reviews, we believed conducting a comprehensive scoping review was necessary.

## Objective

The overall objective of our scoping review was to answer the question “what is known in the literature about CVD management experiences, programs, interventions, and/or recommendations/guidelines specifically for people experiencing homelessness?”. We sought to synthesize the experiences and perspectives of people experiencing homelessness and healthcare teams and, where reported, to document the successes and limitations of delivering CVD care to the homeless population. This review is meant for a broad audience of healthcare providers, health services researchers, and community organizations to understand what knowledge gaps still exist and where we should go next.

## Methods

We performed a scoping review to combine the findings of qualitative and quantitative studies in a single review to address the same overlapping or complementary questions and identify knowledge gaps. We followed the PRISMA Extension for Scoping Reviews checklist and the Arksey and O’Malley 5-Stage Scoping Review Framework [18].

### Lived experience participation

We developed the review protocol and drafted the manuscript in consultation with people with lived experience of homelessness.

### Search strategy

In March 2021, an information specialist at Unity Health Toronto completed database searches in Ovid Medline, Embase, PsychINFO, CINAHL, Web of Science (Science Citation Index), Social Sciences Index, Conference Proceedings Citation Index – Science, Conference Proceedings Citation Index – Social Science & Humanities, Emerging Sources Citation Index), the International Bibliography of the Social Sciences, the Cochrane Central Register of Controlled Trials and Cochrane Database of Systematic Reviews. We translated the search strategies for each database and used comprehensive sets of search terms for *homelessness* (e.g., unstable housing, underhoused, emergency shelter, etc.), *cardiovascular disease* (e.g., heart failure, coronary artery disease, hypertension, etc.), and *programs* (e.g., intervention, self-management, treatment, etc.). The search was limited to English language with no date or geographic limitations. The search was then updated/refreshed in December 2023. See Additional File 1 for the sample Medline Search Strategy. We identified additional studies through hand-searching numerous scientific journals based on their past published content, the first 20 pages of a Google Scholar with search terms, reviewing reference lists and citations of eligible studies, and various national and international grey literature sources known by study team members and collaborators. See Additional File 2 for the Grey Literature Search Strategy.

### Eligibility

All study designs were included, including conference proceedings, study protocols, reviews, perspective pieces, and editorials. Articles were included if they reported lived experiences and perspectives, programs, interventions, and/or guidelines/recommendations for tailored CVD prevention, treatment, or management for adults experiencing homelessness, or who have recently experienced homelessness within the last three years. It was acceptable for studies to include non-homeless groups if the primary population of the study was homeless. CVD included coronary artery disease, arrhythmias, structural heart disease, heart failure, hypertension, and infective heart conditions (e.g., endocarditis). Studies were excluded if the primary study population was homeless youth (< 18 years of age), the study focused on smoking or dietary interventions without specification for CVD, or an abstract was duplicated with the same title/author as a full-text article.

### Screening, extraction, and synthesis

Two reviewers independently screened all titles/abstracts retrieved by the search strategy in Covidence software, followed by full text assessment. After final inclusion, we used a standardized data extraction form in Excel. Data extraction for all studies included: title, author, journal, year, geographic location, study objective/aim, study population, study design/method, and main findings which could be separated into 1) experiences and perspectives of people experiencing homelessness and healthcare providers, 2) programs or interventions (description/characteristics, development, outcomes, strengths/limitations, evaluation, outcome), and 3) guidelines/recommendations for clinical practice and healthcare delivery. Given the heterogeneity and types of articles found, we did not do a meta-analysis. The descriptive data was analyzed using conventional content analysis. The codes were organized into main thematic groups to summarize the literature.

## Results

### Search and study characteristics overview

**Fig. 1:**
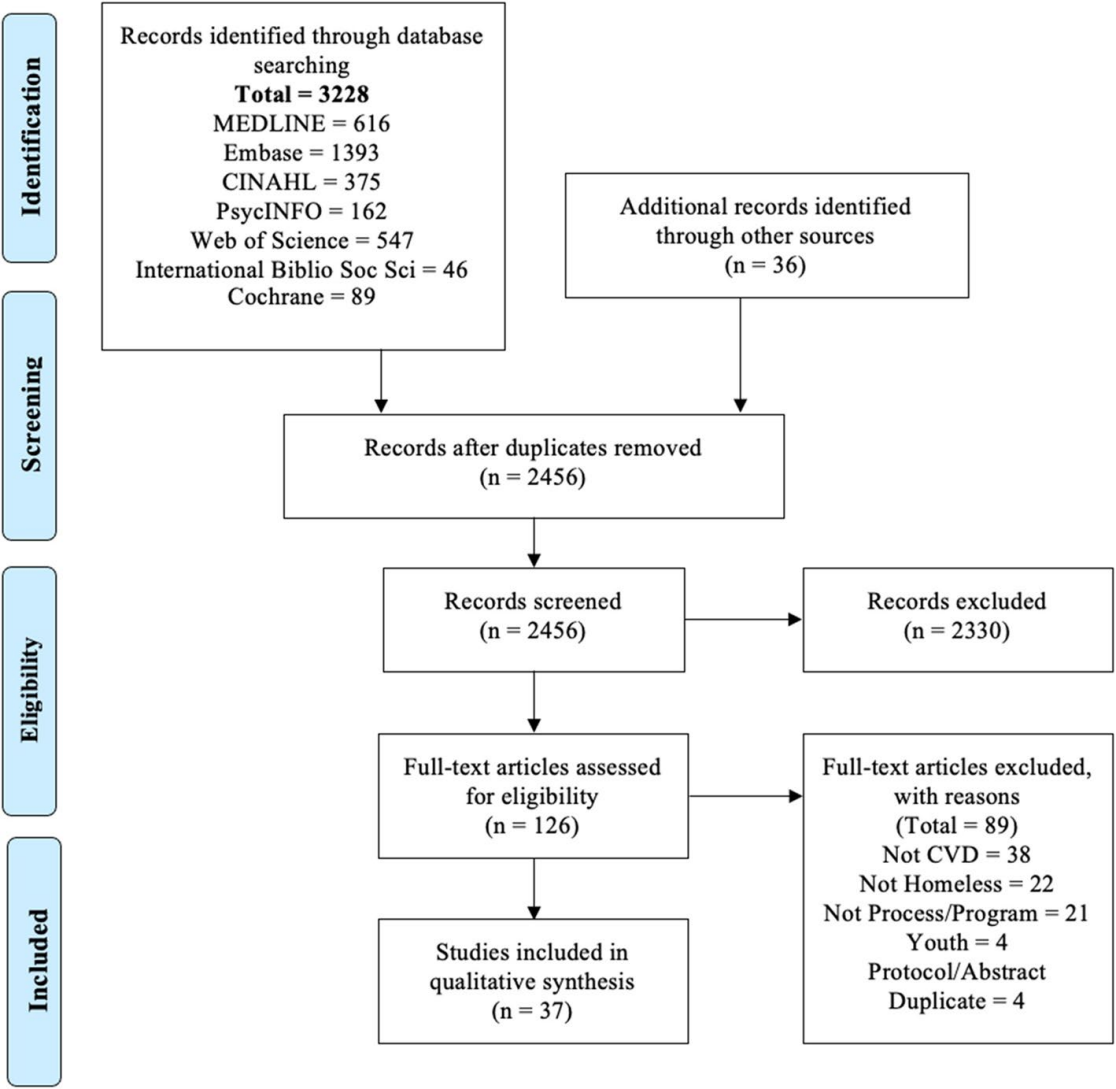
PRISMA Flow Chart

We identified 3228 records through our database search and 36 through our hand-search. After de-duplication, 2456 were screened, and 126 full texts were reviewed. We identified 37 articles that met inclusion criteria. See PRISMA Diagram in Fig. 1.

Of the 37 articles, most work was done recently (post-2015). Most studies were conducted in the USA [19], while others were done in Canada [5], the UK [5], Australia [2], and Denmark [1]. Most studies focused on CVD in general, while some studies focused specifically on hypertension [6], heart failure [4], and endocarditis [1]. We found a wide range of methods used including literature reviews, mixed methods studies, and perspective pieces. Most articles discussed recommendations from their own study results/reviews. We identified seven CVD-specific programs/interventions. Importantly, we did not find any programs or interventions that exist in our home of Canada. Nine studies included perspectives from people experiencing homelessness. Only three studies discussed engaging people experiencing homelessness in the study or program design. See Table 1 for a summary of the articles included.

**Table 1:** Summary of included articles

First Author	Year & Country	Journal	Title of Paper	Study Design	Study Goal, Objective, or Intervention
Albertson [20]	2021, USA	Journal of American Pharmacy Association	*Implementation of primary care clinical pharmacy services for adults experiencing homelessness*	Retrospective Chart Review	To determine the clinical impact and sustainability of implementing clinical pharmacy services in a weekly half-day collaborative multi-disciplinary clinic for adults experiencing homelessness, focused on independent cardiovascular risk reduction and disease management. To evaluate the type and frequency of the interventions made (e.g., medication review, patient education, tobacco cessation, insurance navigation, etc.), and to describe the demographics of patients receiving the services and their clinical outcomes for hypertension (blood pressure), obesity (weight), diabetes (HbA1c), and tobacco use disorder (cessation rate).
Asgary [21]	2016, USA	Annals of Family Medicine	*Rates and predictors of uncontrolled hypertension among hypertensive homeless adults using New York City shelter-based clinics*	Retrospective Chart Review	To study the rates, predictors, and barriers to blood pressure control in homeless hypertensive adults in New York shelter-based clinics.
Baggett [9]	2018, USA	Journal of American College of Cardiology	*Cardiovascular Disease and Homelessness*	Review	To present practical, patient-centred strategies and management suggestions for addressing challenges of CVD homeless patients and an integrated framework for understanding the basis of CVD disparities in homeless people, with a main focus on coronary artery disease prevention, diagnosis, and treatment.
Banerjee [22]	2023, UK	European Heart Journal Supplement	*Cardiovascular disease screening in homeless individuals – a feasibility study*	Feasibility Study (Abstract)	To investigate feasibility of dedicated mobile CVD screening for homeless individuals; and to estimate prevalence of risk factors and burden of CVD.
Bardsley [23]	2012, AUS	Cardiac Society of Australia and New Zealand	*Targeting a hard to access population - heart failure and homelessness in Sydney*	Descriptive Qualitative (Abstract)	To describe St. Vincent’s chronic heart failure disease management program that works with a local homeless hostel to make an outreach clinic providing multi-disciplinary HF care to complex patients with multi-morbidity.
Bark [24]	2023, UK	Open Heart	*Integrated approach to cardiovascular disease in people experiencing homelessness: a qualitative study*	Qualitative (Focus Groups)	To understand and make recommendations to improve CVD care in homeless populations through lived and professional experience.
Bearnot [19]	2019, USA	Journal of Substance Abuse Treatment	*Experiences of care among individuals with opioid use disorder-associated endocarditis*	Descriptive Qualitative (Interviews)	To elucidate the prior experiences of care for patients with opioid-use disorder-associated infective endocarditis and the healthcare providers who deliver that care. Of 11 patients, 6 were unstably housed.
Beggs [25]	2016, USA	Journal of Health Care for the Poor and Underserved	*Effectiveness of pharmacy student-led health education in adults experiencing homelessness*	Descriptive Quantitative (Survey)	To assess the effectiveness of pharmacy student-led education on chronic disease (asthma, diabetes, hyperlipidemia, hypertension, smoking cessation) in adults experiencing homelessness in one-hour sessions.
Brandt [26]	2023, USA	JACC State-of-the-art reviews	*Assessing and addressing social determinants of cardiovascular health*	Review	To cover five domains of social determinants of health (economic stability, education, health care access and quality, social and community context, and neighbourdhood and built environment). They discuss each SDOH within the context of cardiovascular disease, how they can be assessed by clinicians and within health care systems and key strategies for clinicians and health care systems to address them.
Brickner [27]	1993, USA	Bulletin of the New York Academy of Medicine	*Providing health services for the homeless: a stitch in time*	Overview	To discuss how healthcare services are designed and offered at homeless shelters for those with hypertension, TB, and HIV.
Chum [28]	2020, CAN	Journal of American Heart Association	*Effect of a housing intervention on selected cardiovascular risk factors among homeless adults with mental illness: 24-month follow-up of a randomized controlled trial*	Randomized Controlled Trial	To determine whether a Housing First intervention to give housing stability to homeless adults with mental illness had an effect on participants’ selected CVD risk factors. The intervention was for high needs (Housing with support from assertive community treatment (ACT)) or moderate needs (housing with intensive case management). They also had access to an onsite primary care clinic (overall health, not specifically cardiovascular).
Dickens [29]	2017, USA	American College of Cardiology	*Homelessness and heart failure: a novel prescription (abstract)*	Case study (Abstract)	To present a case study of a homeless man with heart failure.
Dickens [30]	2019, USA	Journal of Cardiovascular nursing	*Perceived stress among patients with heart failure who have low socioeconomic status: a mixed-methods study*	Mixed Methods (Interviews, Perceived Stress Scale Survey, and Self-Care of HF Index Survey)	To describe the influence of stress and social determinants of health on self-care in patients with heart failure who have low SES and were readmitted within 120 days of a HF exacerbation – 4 participants of 35 were homeless.
Fazel [31]	2014, UK	Lancet	*The health of homeless people in high-income countries: descriptive epidemiology*,* health consequences*,* and clinical and policy recommendations*	Overview	To provide recommendations to improve the surveillance of mortality in homeless individuals, including a specific section on cardiovascular and metabolic disease.
Gabrielian [32]	2013, USA	Medical Care	*Chronic disease management for recently homeless veterans: a clinical practice improvement program to apply home telehealth technology to a vulnerable population*	Mixed Methods (Chart review, Survey, Interview)	To describe the early phases of applying and testing an existing Veterans Affairs HIT-care management program, Care Coordination Home Telehealth (CCHT) to recently homeless veterans, assess program acceptability, and inform future quality improvement. They are in-home messaging devices to provide health education and daily questions about clinical indicators from chronic illness care guidelines (hypertension, CHF, COPD, diabetes, obesity).
Groton [33]	2020, USA	Public Health Nursing	*“You got to eat*,* but then what you are eating*,* it’s going to kill you”: Living with hypertension while experiencing homelessness*	Descriptive Qualitative study (Focus Groups)	To explore the barriers and facilitators of self-management of hypertension while experiencing homelessness.
Jones [34]	2009, CAN	Current Cardiology Reviews	*Cardiovascular Disease Risk Among the Poor and Homeless – What We Know So Far*	Review	To review the available literature detailing the relationships between poverty, homelessness and cardiovascular disease. Secondarily, some of the more current efforts aimed at reducing disparities in CVD care of the poor and homeless are discussed.
Karashin [35]	2018, USA	Institute Report	*Street Medicine Clinical Guidelines: Hypertension*	Clinical Guidelines Report	To create clinical guidelines to diagnose, manage, and prevent hypertension in unsheltered homeless individuals on top of general clinical guidelines for the general population.
Kinchen [36]	1991, USA	American Journal of Public Health	*Hypertension management in health care for the homeless clinics: results from a survey*	Descriptive Quantitative (Survey)	To survey healthcare providers that had health care clinics for the homeless and understand how they manage hypertension in this population.
Klein [37]	2015, USA	Medical Clinics of North America	*Care of the homeless*	Discussion Paper	To discuss general strategies when caring for homeless patients, highlight practical tips for addressing common clinical syndromes, and discuss the unique needs of vulnerable homeless subpopulations.
Lane [38]	2020, UK	Heart	*Homelessness: a cause of the causes’ of cardiovascular disease?*	Editorial	To provide comments on the importance of Al Shakarchi *et al’s* systematic review entitled “cardiovascular disease in the homeless versus housed individuals: a systematic review of observational and interventional studies”.
Lee [39]	2005, CAN	Circulation	*Risk factors for cardiovascular disease in homeless adults*	Mixed Methods (Interviews, Blood Measures)	To examine cardiovascular risk factors in a representative sample of homeless adults and identify opportunities for improved risk factor modification.
Liauw [40]	2021, CAN	Canadian Journal of Cardiology	*Clinical presentation and outcome of patients experiencing homelessness presenting with ST-Segment Elevation*	Retrospective Cross-Sectional Study	To curate the baseline characteristics, clinical management, and in-hospital outcomes of people experiencing homelessness presenting with ST-elevation myocardial infarction at a PCI-capable STEMI network inner-city hospital.
Maness [41]	2014, USA	American Family Physician	*Care of the homeless: an overview*	Overview	To give an overview of homelessness health, including blood pressure, cholesterol, and diabetes goals.
Maqsood [11]	2020, USA	Clinical Medicine Insights: Cardiology	*Cardiovascular issues among homeless people: an issue that needs attention*	Perspective	To discuss the issues associated with cardiovascular disease treatment, outcomes, and future directions for homeless persons.
Moczygemba [42]	2013, USA	Research in Social and Administrative Pharmacy	*A qualitative analysis of perceptions and barriers to therapeutic lifestyle changes among homeless hypertensive patients*	Qualitative (Focus Groups)	To identify homeless patients’ knowledge and perceptions of hypertension and lifestyle changes and understand the barriers to implementation of lifestyle changes.
Murphy [43]	2015, USA	Journal of American Association of Nurse Practitioners	*Improving cardiovascular health of underserved populations in the community with Life’s Simply 7*	Mixed Methods (Health Measures Survey and Self-Report)	To collect the pre-program and post-program health data at two inner city community sites of a nurse practitioner-led initiative to improve cardiovascular health using American Heart Association’s Life’s Simple 7 and My Life Check (MLC) tools, including pre- and post-health screening, coaching sessions, teaching materials, motivational interviewing, and goal setting.
Nanjo [44]	2020, UK	European Heart Journal	*Prevalence*,* incidence*,* and outcomes across cardiovascular diseases in homeless individuals using national linked electronic health records*	Retrospective Cohort Study	To investigate prevalence, incidence and outcomes across a range of specific cardiovascular diseases among homeless individuals to start to address tailored solutions.
Nayyar [45]	2015, CAN	Canadian Journal of Cardiology	*Cardiovascular health issues in inner city populations*	Review	To provide a narrative outline of the burden of cardiovascular disease in inner city neighbourhoods of high-income countries, possible mechanisms leading to this inequity, and interventions to improve the cardiovascular health of inner-city communities, including homeless individuals.
Nyembo [46]	2023, USA	Current Cardiovascular Risk Reports	*Homelessness*,* race/ethnicity*,* and cardiovascular disease: a state-of-the-evidence summary and structured review of race/ethnicity reporting*	Review	To examine the intersection of cardiovascular disease, race/ethnicity, and homelessness/unstable housing to understand the deep roots of structural racism in housing and healthcare.
Palmer [47]	2018, USA	Homeless Older Adults	*Cardiovascular disease in homeless older adults*	Review	To review the literature on cardiovascular disease in homeless older adults and provide practice adaptations for clinicians and teams.
Pendyal [48]	2021, USA	Heart & Lung	*When you’re homeless they look down on you: a qualitative*,* community-based study of homeless individuals with heart failure*	Qualitative (Interviews)	To identify ways in which homelessness may impede successful self-management of heart failure and engagement with the healthcare system for lived experience perspective.
Randers [49]	2012, DEN	European Journal of Applied Physiology	*Short-term street soccer improves fitness and cardiovascular health status of homeless men*	Non-Randomized Trial	To determine the effect of street soccer and fitness training on the cardiovascular health of homeless men. It was 12 weeks of small-sided street soccer (2-3x per week) where they measured pre- and post- maximal oxygen uptake, total fat mass, and low-density lipoprotein cholesterol in a group of 22 homeless men when compared with controls.
Rao [50]	2022, AUS	Heart, Lung and Circulation	*Cardiovascular disease in Australians experiencing homelessness*	Narrative Review	To explore how a combination of inadequately managed traditional and non-traditional cardiovascular risk factors, along with several personal, practical and relationship challenges in the health system, have created unique barriers in diagnosis and management of cardiovascular disease in Australians experiencing homelessness to propose a collaborative strategy.
Strehlow [51]	2009, USA	Independent Report	*Adapting your practice: treatment recommendations for homeless patients with hypertension*,* hyperlipidemia*,* and heart failure*	Report	To provide treatment recommendations for homeless patients with hypertension, hyperlipidemia, and heart failure.
Wadhera [10]	2020, USA	JAMA Internal Medicine	*Disparities in care and mortality among homeless adults hospitalized for cardiovascular conditions*	Retrospective Cross-Sectional Study	To evaluate differences in intensity of care and mortality between homeless and non-homeless individuals hospitalized for cardiovascular conditions (e.g., acute myocardial infarction, stroke, cardiac arrest, or heart failure).
Wright [52]	2021, USA	Mayo Clinic Proceedings	*Homeless With a Heart Attack in* *America: A Double Whammy*	Editorial	An editorial on a paper by Balla et al. that reports on the effect of homelessness on outcomes in patients with acute myocardial infarction.

### Main themes

We have synthesized the articles’ findings into main themes. We start with the reported challenges and barriers faced by people experiencing homelessness and their providers with CVD management and care delivery. We then summarize seven existing international programs and interventions for CVD care for the homeless population, including their specific outcomes and learnings, and general considerations for program building. Finally, we summarize the literature’s recommendations and potential solutions of CVD management in the homeless community at the patient encounter level, clinical organization level, and systems level.

#### Challenges and barriers

**Fig. 2:**
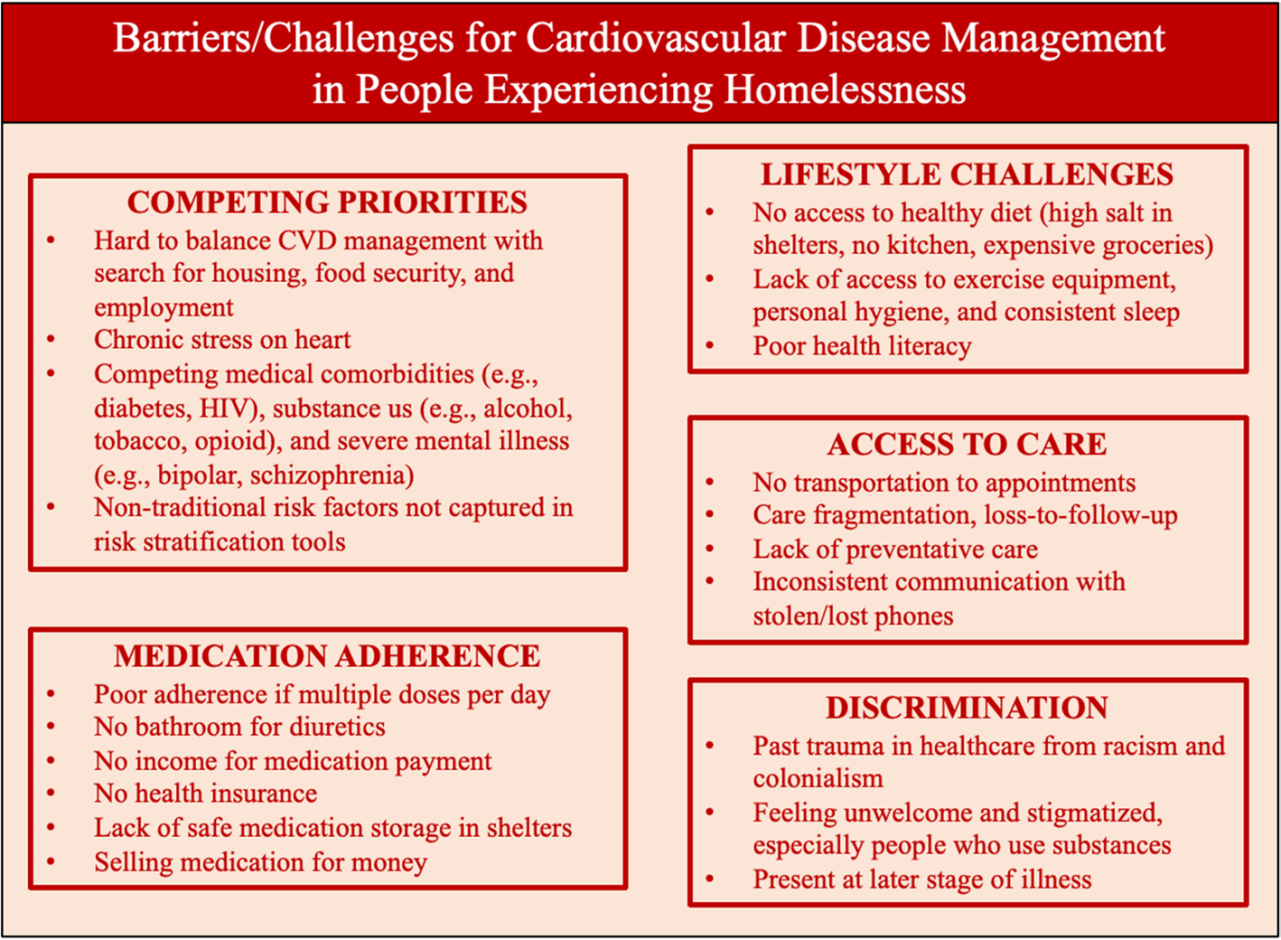
Summary Figure of Barriers and Challenges of CVD Management for People Experiencing Homelessness

Various studies touched on the experiences, challenges, and barriers of CVD prevention, management, and care delivery for the homeless population. These are reported in the following sub-themes: competing priorities, lifestyle challenges, medication adherence, access to care, and discrimination. See Fig. 2 for a Summary Figure of challenges and barriers.

### Competing priorities

A major theme was the competing priorities that people experiencing homelessness must face in their daily life - including looking for housing, food security, and employment - that make CVD management challenging to balance. An emerging theme stemming from this was the ongoing chronic stress of people experiencing homelessness that affects organizing daily life necessities and the physiologic load this puts on the heart [9, 30, 33]. The literature reported many people experiencing homelessness have competing and uncontrolled medical comorbidities (e.g., diabetes, COPD, HIV, Hepatitis C, among others), substance use disorders (e.g., alcohol, tobacco, cocaine, crystal meth, heroin, fentanyl, among others), and/or severe mental illness (e.g., Bipolar Disorder, schizophrenia, among others) that impact ability to focus on CVD management or that further worsen CVD outcomes. Barriers are further created when these non-traditional risk factors are not captured in risk stratification tools and calculators [39, 40].

### Lifestyle challenges

Another common theme was people experiencing homelessness do not have access to healthy diets for dietary modification of CVD, reported in many ways: high-salt content in shelters with limited food choices, a lack of kitchen to prepare their own healthy meals, and the high prices and poor locations of healthy food and grocery stores. Other lifestyle challenges included lack of access to exercise equipment, lack of access to showering and hygiene facilities, and lack of a consistent or comfortable bed for routine sleep. An emerging theme was that people experiencing homelessness have lower health literacy posing a barrier to CVD management. For example, people experiencing homelessness may have a mixed understanding of how lifestyle affects hypertension with many people being familiar with diet and smoking recommendations, but less familiar with the impacts of exercise, alcohol, and caffeine [42].

### Medication adherence

Alongside competing priorities, a commonly reported challenge for people experiencing homelessness and CVD management was medication adherence, especially with medication that has multiple doses per day as they lack consistent routine, requires specific access (e.g., readily available bathroom access for diuretic use for heart failure), or requires storage (e.g., refrigerator, or locked storage to avoid getting stolen in shelters). Another major theme was a lack of income to pay for medication or no health insurance (both in countries with universal health coverage and not). A less commonly reported barrier was having to sell their medication for substances or bus fare [9].

### Access to care

For access to healthcare, a commonly reported challenge was not having transportation to get to medical appointments or to the pharmacy. This was especially important when thinking about CVD diagnoses requiring multiple measurements and consultations over time (e.g., many BP readings, ECG followed by echo) which people experiencing homelessness often cannot access or commit to [9]. A recurring theme was people experiencing homelessness often do not have a continuous source of care, causing care fragmentation, loss-to-follow-up and lack of preventative care in both primary and specialty care settings. Some people experiencing homelessness have reported difficulty in consistent communication with their providers due to lost or stolen phones [9, 32].

### Discrimination

A major theme was people experiencing homelessness have experienced past trauma in the healthcare system and experience ongoing discrimination, stigmatization, and/or overall feeling of unwelcomeness. This included discrimination based purely on being homeless, being racialized, having refugee status, or being in the LGBTQ + community, among others. In turn, this often means people experiencing homelessness are presenting at a later stage of disease, or not receiving appropriate discharge supports without divulging their housing status [24, 33, 48]. It was an especially prominent theme for people presenting late with infective endocarditis, which is often seen as an injection drug use condition where they face stigma for returning to drug use after hospitalization [19].

#### Existing programs and interventions

See Table 2 for a summary of seven programs/interventions in the international literature for CVD management and care delivery for the homeless community.

**Table 2:** Existing CVD programs and interventions

Program/ Intervention	Description	Outcomes/Learnings
Primary care clinical pharmacy services [20]	They added a pharmacist with an expanded skillset to a weekly half-day collaborative primary care clinic for people experiencing homelessness, to focus on independent cardiovascular risk reduction and disease management. The pharmacy services included medication access/storage (safety and refrigeration), medication changes and refills, conversations about goals of care, discussions on lifestyle changes and interventions, insurance coverage navigation, provision of bus passes to and from monthly pharmacy medication refills, and medication vouchers for patients with unaffordable co-payment.	Over 8 months, the pharmacist had 28 encounters for 14 unique patients and made a high mean of 4 clinical interventions per patient encounter such as medication review, patient education, medication regime optimization, and tobacco cessation, among others. Clinical outcomes (blood pressure, weight, HbA1c, tobaccos use) remained stable during this period. This work highlighted the importance of multi-disciplinary teams at a one-stop place (physicians, psychiatrists, nurse practitioners, dieticians, nurses, social workers, care coordinators), that is tailored to the unique needs of the homeless population, which includes the pharmacist in this group with an expanded skillset, especially in these low-resource settings.
Chronic heart failure disease outreach management program that works with a local homeless hostel [23]	No specific information on how this program works was provided; only that this outreach initiative exists to provide multi-disciplinary heart failure care to complex patients with multi-morbidity.	Seven patients regularly presented to the hostel for the last five months, and there was initiation and up titration of cardiac medication. They reported heart failure symptoms improved, there were no heart failure-related hospital admissions for these patients during this period, and there were referrals to other health professionals.
Nurse practitioner-led program using American Heart Association’s Life Simple 7 and My Life Check Tools [43]	A nurse practitioner used AHA’s Life’s Simple 7 and My Life Check Tools, including pre -and post-health screening, coaching sessions, teaching materials on health factors, aLS7 health guides for customized recommendations for positive behaviours to improve cardiac health, and logs to document physical activity, dietary intake, smoking habits, blood sugar, weight, and blood pressure. They used evidence-based techniques of motivational interviewing, goal setting, and self-monitoring to develop a trusting partnership to tailor the intervention to the patient. Their participants included older adults and women experiencing homelessness.	Eight older adults completed the program with a 37.1% increase on average in their My Life Check (MLC) Score. In comparison, ten women experiencing homelessness completed the program with a 9.3% decrease in MLC score, therefore favouring benefits to older adults rather than homeless individuals. They discuss these benefits may not have been seen in people experiencing homelessness given not understanding their social contexts and that we can learn from failed interventions.
Pharmacy student-led education gams [25]	Five groups of pharmacy students each developed three Bingo games focused on a primary care health topic (asthma, diabetes, hyperlipidemia, hypertension, smoking cessation). Each group delivered the content via a one-hour class offered to adults experiencing homelessness at a local outreach organization. The questions focused on basics of disease, anatomy, statistics, medicines, diet, lifestyle, environmental concerns, misconceptions. Each answer had a PowerPoint slide teaching point.	There were 37 surveys completed about the Bingo game. Survey results showed that it increased knowledge in the homeless population and that participants would attend again. The study concludes that this is an effective way to increase knowledge and teaching in this population.
Organized street soccer [49]	A 12-week program of organized small-sided street soccer games for 60 min, 2–3 times per week in the homeless community to improve cardiac health with post-testing procedures.	Twenty-two men experiencing homelessness were enrolled into the program, compared to ten controls not enrolled. They found that the soccer intervention improved maximal oxygen uptake, total fat mass, and low-density lipoprotein cholesterol, as well as improvements in endurance and intermittent exercise capacity. For homeless participants who had high cholesterol and diastolic blood pressure at the start of the intervention, there were also decreases in these measurements.
Home telehealth program for homeless veterans [32]	The program applied an existing Veterans Affairs HIT-care management program called Care Coordination Home Telehealth to homeless veterans. They were given in-home messaging devices to provide health education and daily questions about clinical indicators from chronic care guidelines, by nurses. Chronic diseases included hypertension, congestive heart failure, COPD, diabetes, obesity, and depression. Daily questions for cardiac illness included weight and blood pressure measurements. Red flag values or nonadherence alerted nurses for telephone follow-up. They also received peer support from previously homeless veterans with health conditions.	Fourteen veterans experiencing homelessness participated, 10 of them with peer support and 4 without. Of 14, 50% had hypertension, 80% dyslipidemia, 0% CHF, and 30% coronary artery disease. There was overall satisfaction with the program and for peer support to help social isolation. They stated the technology gave information they would not get elsewhere, explained clinical symptoms well, felt like someone was paying attention, and was an avenue for monitoring and intervening early. On the other hand, they stated the device was impersonal and felt mundane asking the same questions. Some had distrust of technology and poor adherence. Of note, many refused to enroll from the start.
Mobile CVD screening [22]	Co-developed and implemented with patients, a 15-minute CVD assessment for homeless individuals as an add-on service to their already-established Find&Treat service (a peer-led, multi-disciplinary outreach team across London UK) at routine scheduled visits in homeless hostels. There was a questionnaire (demographics, past medical history, symptom data) and clinical assessment (blood pressure, ABPI, cholesterol, glucose, HBA1c, and ECG for atrial fibrillation)	Of 656 people approached, they screened 524 (79.9%) in a four-year time span, mostly male and white. The majority smoked and 15% had previous stroke/MI. By screening, 25% had hypercholesterolemia, 12.3% atrial fibrillation, and 22% diabetes. One third had a 10-year QRISK score of > 10%. They concluded a simple pragmatic CVD assessment for homeless individuals was feasible and acceptable, and they showed gaps in both diagnosis and prevention. CVD morbidity and mortality could be addressed by integration of CVD screening into existing inclusion health outreach frameworks.

In addition to the learnings from the programs/interventions described, the overall literature reported on important considerations for future development of CVD programs for the homeless community. A major theme was engaging key stakeholders and community leaders in the early planning and implementation of future programs to ensure understanding of the social context of the population being served. This included partnering with charitable organizations, community health centres, housing organizations, and those with lived experience. Another major theme was that future CVD programs must take a multi-disciplinary approach, including multi-level government support, community support workers, and specialists in psychiatric care. This included specifically having cardiac surgeons and addictions teams included in endocarditis programs [19, 40]. An emerging theme was prioritizing flexibility, creativity, and innovative ways to adapt to the homeless setting, including digital health solutions [32, 40]. Finally, another emerging theme was for program pilots to move forward from feasibility studies into economic and scalability studies with full evaluation [9, 14, 24].

#### Recommendations and solutions

**Fig. 3:**
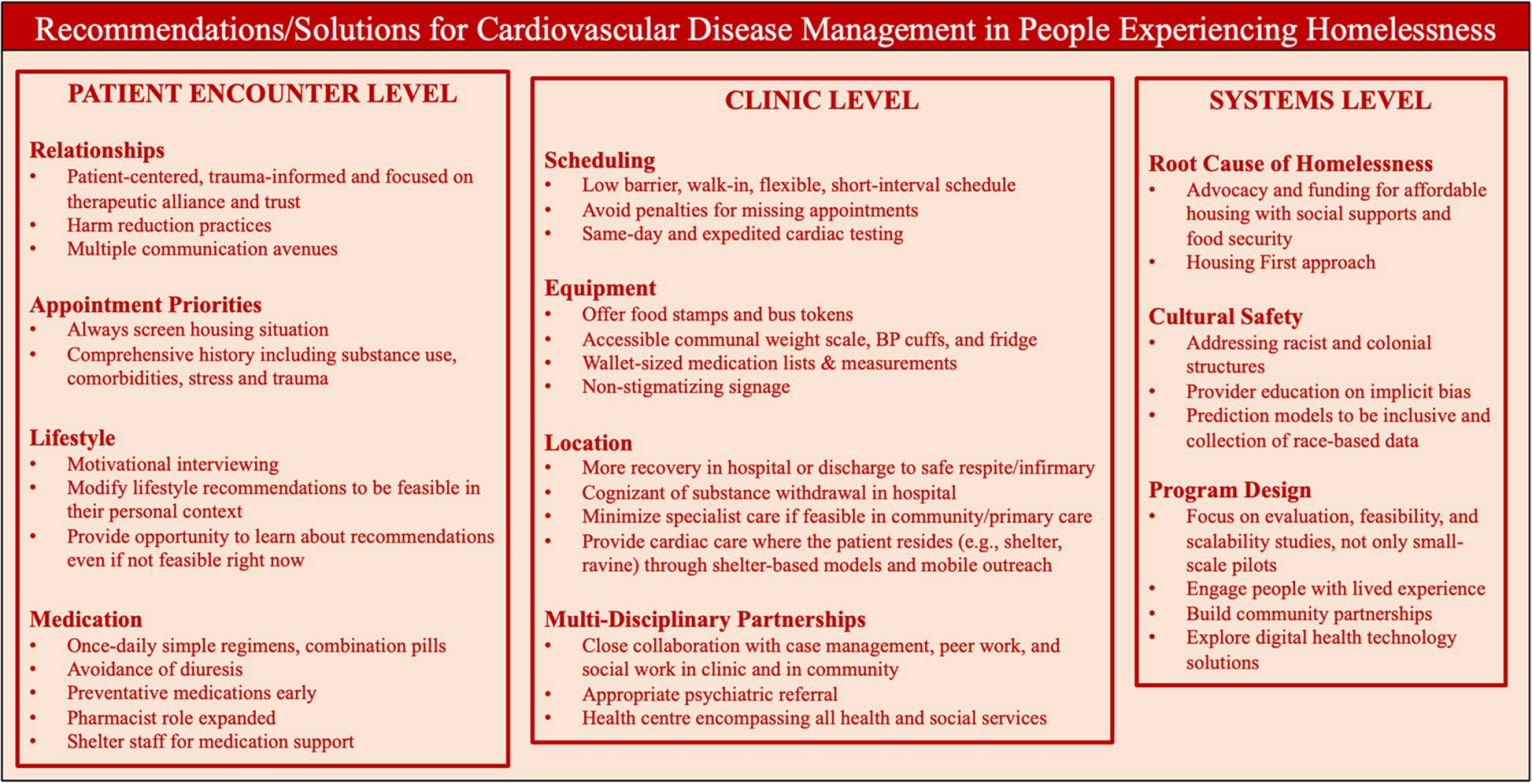
Summary Figure of Recommendations and Solutions of CVD Management for People Experiencing Homelessness

Various studies touched on practical recommendations and possible solutions for CVD prevention and management in the homeless community. Some recommendations and solutions stemmed specifically from a reported barrier, and others did not. We have organized these recommendations and solutions into the *patient encounter level* (relationships, appointment priorities, lifestyle, and medication), *clinic organization level* (scheduling, equipment, location, and multi-disciplinary partnership), and *systems level* (root cause, and cultural safety). See Fig. 3 for a Summary Figure of recommendations/ solutions.

## Patient encounter level

### Relationships

A major theme when reporting recommendations for CVD care in the homeless community was that relationships between people experiencing homelessness with CVD and their care provider (clinical, social, etc.) must be patient-centred, trauma-informed, and focused on a therapeutic alliance that is founded on trust and dignity. While providers should be aware of the numerous non-traditional risk factors in this population such as substance use and severe mental illness, they must be discussed with care. An emerging theme was that this recommendation is especially important for patients with endocarditis and a harm reduction approach must be prioritized (e.g., helping reduce substance use or use drugs more safely [19]. Reporting about patient-provider relationships also commonly included having many communication avenues with the patient and people in their circle that they trust (e.g., case worker, street family).

### Appointment priorities

In a clinic appointment, a major theme was that the provider should always inquire about living situation/housing status up front and inquire about what supports they have in acquiring housing. A common screening tool noted was Medicare and Medicaid 10-item screening tool by Billioux et al. in 2017 in the USA [53]. Among others, Baggett and Strehlow particularly believe the following are the key aspects we should discuss when having a CVD appointment with people experiencing homelessness: housing status, basic needs such as food security, undiagnosed/untreated hypertension, uncontrolled diabetes, lipid-lowering medications, smoking, alcohol use, cocaine/amphetamine use, IV drug use, chronic stress and trauma, HIV, PTSD, depression, and schizophrenia [9, 51]. A secondary theme was the idea that any measurements done at the visit (e.g., blood pressure) need a detailed history hours prior to the measurement (e.g., substance use) [35].

### Lifestyle

In terms of lifestyle measures, it was a common theme for providers to use motivational interviewing techniques to understand people experiencing homelessness’ competing priorities and what lifestyle changes would be personally feasible at this time. Another recurrent theme was for providers to modify their lifestyle recommendations to what the patient can do (e.g., educating a patient with heart failure to be able to notice leg edema rather than doing daily weights without an accessible weight scale [26]). An emerging theme also recommended to focus on stress management techniques [30, 41]. A tension in the literature arose where some providers believed lifestyle interventions (e.g., smoking cessation, diet plans) are not a recommended priority given people experiencing homelessness’ competing priorities - “*Although therapeutic goals between homeless and housed individuals are similar*,* the means chosen to achieve them are often not”* [36] - while other providers and people experiencing homelessness believed these were still a priority for people to decide on their own what they are capable of, or for future use when stably housed. Moczygemba particularly focused on a greater education emphasis on immediate benefits of controlling blood pressure which can be a silent disease, and on serious cardiac arrest symptoms to warrant going to hospital [42].

### Medication

Practical solutions for giving medications were a common theme in the literature, including once-daily medications, medications with simple regimens, combination pills, non-refrigerated medications, and medications that do not need to be taken with food given food insecurity. Other recurring recommendations included prescribing calcium channel blockers for heart failure management - rather than ACE-inhibitors or diuretics - due increased urinary side effects, encouraging early initiation of statins for dyslipidemia, and pharmacotherapy for smoking cessation. A smaller theme was ensuring substance users understand the importance of taking medications even when actively using illicit substances and discussing contraindications [51]. Emerging themes were having the pharmacist’s role expanded for people experiencing homelessness to include more medication management and risk reduction [9, 20, 42], and having shelter staff and nursing staff where people experiencing homelessness reside for medication reminders and management [41, 47].

## Clinic level

### Scheduling

In almost all studies in this review, there was a recommendation of clinics needing to offer walk-in appointments, flexible scheduling, and avoiding punishment for missing appointments. A major theme was avoiding loss-to-follow-up by having shorter intervals between appointments/prescriptions, same-day testing, and urgent/expedited stress testing. Less commonly listed recommendations were giving incentives for patients to return and bringing patients to the clinic room immediately from the waiting room to avoid self-consciousness of hygiene [9, 51].

### Equipment

A recurrent recommendation was for clinics, health centres, and community organizations (e.g., shelters, drop-in centres) to be well-equipped for people experiencing homelessness with CVD: offer food stamps for healthier food options and bus tokens for transportation to clinics, have a weight scale and/or blood pressure cuff available for public and free access in the waiting room, create wallet-sized cards with medication lists and measurements, offer pre-filled medication boxes that are weather-resistant to help take daily medications, have an accessible fridge for storing temperature-sensitive medications, offer extra pillows for those with heart failure that need elevation, and have non-stigmatizing signage present in the building [9, 31, 33, 35, 36, 51].

### Location

An over-arching theme was that CVD management for the homeless community should happen across the continuum of the healthcare system with primary care risk factor management and chronic care management in the community and acute and specialized management with specialty care. An emerging theme was to provide care where the patient resides (e.g., shelter, respite, park) through shelter-based healthcare or mobile outreach teams, minimizing referrals to specialists if the care can be provided in the community [9, 22, 24, 36]. It was also commonly reported that if a person experiencing homelessness with CVD is admitted to hospital, there should be advocacy to discharge them to respite care or an infirmary, rather than the streets, including advocacy for increased funding for these institutions. Bearnot et al. highlighted that providers should be aware of excessively long hospitalization (e.g., 6 weeks of IV antibiotics for endocarditis) that providers may see as positive for treatment purposes and counselling, whereas patients perceive it as potentially injurious for substance withdrawal and pain management [19].

### Multi-disciplinary partnerships

Similar to programs and interventions needing to focus on multi-disciplinary teamwork, a major theme in the literature was for healthcare clinics to have close collaboration with multi-disciplinary services in the health and social service sectors, such as case management, social work, grassroots cultural organizations, and medication assistance programs, an example being the Homeless Veteran Services in the USA [9]. A commonly proposed solution was a community health centre or equivalents where all services were in one spot, a “one-stop-shop”. Another major theme was ensuring referring to appropriate concurrent psychiatric or substance use supports for people experiencing homelessness with CVD. Al-Shakarchi et al. concluded that “*integrated care is likely the most viable*,* effective solution for the CVD burden for prevention to management in coordination with other physical*,* behavioral*,* and social needs*” [14].

## Systems level

### Root cause of homelessness

Almost every article reported the major theme of addressing the root cause of homelessness: safe, affordable, supportive housing. People experiencing homelessness with CVD cannot be physically, emotionally, socially, or mentally healthy without first having a stable and secure place to live. This included supportive housing with healthcare support on site for chronic disease. An emerging theme included healthcare institutions providing financial support to housing organizations [26] and advocating and funding for healthier food options in food banks for food security [34, 45, 51]. While the literature largely concurred with these policy recommendations, it is also important to highlight that in a 2020 Canadian randomized controlled trial of Housing First – where people experiencing homelessness are provided community-based housing and supportive services – it unexpectedly did not result in greater improvements in many selected cardiovascular risk factors, compared to those treated as usual [28]. The trial concluded that while housing is necessary to improve the health of people experiencing homelessness, it is not solely sufficient to reduce the high level of CVD risk in this population.

### Cultural Safety

A common theme in the literature was that healthcare institutions must be more culturally safe for people experiencing homelessness and must address structural determinants such as racism and colonialism from the top down. This included healthcare provider education on discrimination/stigma and understanding providers’ own implicit biases. An emerging theme was for governments to provide more funding to high-risk hospitals and institutions who serve people experiencing homelessness and are strong in cultural safety [10]. Nyembo et al. explained that housing is an example of structural racism that results in health inequities for those who are homeless. They used an intersectional approach to understand any compounding challenges for people experiencing homelessness who are Black and Indigenous, with the future recommendation of collecting race-based data with defined categories and reporting standards [46].

## Discussion

This scoping review answered the overall objective of understanding what is known in the literature about CVD prevention and management experiences, programs, interventions, and/or recommendations specifically for people experiencing homelessness with CVD. It included 37 articles, 7 CVD-specific programs/interventions, and a summary of the challenges and barriers to management with recommendations and possible solutions. See Figs. 2 and 3 for respective summary figures.

The literature was largely aligned that people experiencing homelessness have unmet cardiovascular management and care delivery needs, and that there are few practically implemented programs to tackle these unmet needs. This review serves as a call to action to multi-disciplinary primary care, cardiology, and community organizations working with the homeless community, that there is an urgent need of new programs and interventions for context-based CVD management for people experiencing homelessness. While healthcare delivery in the homeless community is often complex and intersectional with many systems level factors that must be addressed upstream [45], we must seek community partnership and lived experience expertise to further understand the context and needs of this community when developing solutions.

### Literature gaps

This review separated challenges/barriers and recommendations/solutions to understand the landscape of the literature. Often, an article would address one or the other. Therefore, we analyzed if there was a correlation between barriers and solutions, or if there was a specific gap in the literature that may be an important next step in future research, program development, and/or clinical practice. For each of the five reported barriers, we correlated the recommendation/solution(s) that were addressing it. The barrier ‘Competing Priorities’ was addressed by *Relationships*,* Appointment Priorities*,* Scheduling*,* Multi-Disciplinary Partnerships*,* and Root Cause of Homelessness.* The barrier ‘Lifestyle Challenges’ was addressed by *Relationships*,* Appointment Priorities and Lifestyle* (an entire recommendation theme to address it). The barrier ‘Medication Adherence’ was addressed by Relationships and *Medication* (an entire recommendation theme to address it). The barrier ‘Access to Care’ was addressed by *Relationships*,* Equipment*,* Location*,* and Multi-Disciplinary Partnerships.* Finally, the barrier ‘Discrimination’ was addressed by *Relationships*,* Root Cause of Homelessness*,* and Cultural Safety.* It is important to note the recommendation *Relationships*, including patient-centred and trauma-informed care based on trust, was a key recommendation throughout the entire body of literature. The Medication Adherence barrier and the Lifestyle Challenge barrier both had specific recommendation themes to address them. Although these five barrier themes continue to be practically unaddressed in our healthcare system, each barrier in the literature at least had recommendations or theoretical proposed solutions to address them.

One large gap, however, was what proposed recommendation or solution was most important for clinicians, researchers, and community organizations to prioritize when thinking about implementing a CVD care pathway in their own health system. This also led to a gap in understanding what proposed recommendations or solutions were most effective and desired for specific contexts, such as geographic location, specific homeless sub-groups, or based on resource availability. These gaps were driven by the literature reporting only seven CVD-specific programs/interventions for people experiencing homelessness. The first reported intervention of expanding pharmacy’s role in a collaborative primary care clinic for people experiencing homelessness [20] addressed barriers Competing Priorities, Lifestyle Challenges, and Medication Adherence. The chronic heart failure disease outreach management program that works with a local homeless hostel [23] addressed Access to Care and Medication Adherence. The nurse practitioner-led program of AHA’s Life Simple 7 [43] addressed Competing Priorities and Lifestyle Challenges. The student-led CVD Bingo game at shelters [25] and the organized street soccer intervention [49] addressed Lifestyle Challenges in health literacy and exercise, respectively. The home telehealth program for homeless veterans [32] addressed Competing Priorities, Access to Care, and Medication Adherence. Finally, the mobile CVD screening program in homeless hostels [22] addressed Access to Care and Lifestyle Challenges. It was positive to see many of these programs and interventions were founded on unmet needs and reported barriers, as well as implemented multi-disciplinary team work to address needs from many health and social directions. It was concerning that none of these programs addressed the barrier of Discrimination; this highlights a barrier for urgent attention.

We also believe these gaps were driven by the fact that these programs and interventions were small-scale pilots (< 15 participants). These program pilots must have full evaluation, feasibility, economic, and/or scalability studies that follow to ensure we have full practical understanding. Additionally, while these program pilots have predominantly been positive, this could be influenced by publication bias. This demonstrates the need to publish failed interventions for others to learn from and grow our body of literature on challenges and solutions to CVD care delivery in the homeless population.

### Adjacent literature

There were various articles not included in this review because the primary study population was not people experiencing homelessness, but they did focus on inner-city and intersectional vulnerable populations. For example, Walton-Moss et al. synthesized community-based CVD interventions among vulnerable populations (e.g., racial minorities, low-income neighbourhoods, among others) [8]. They reported a few small-scale studies of CVD interventions that showed some success in improving cardiovascular outcomes through use of mobile clinics, health coaching, and case management approaches, and a larger-scale study that built a generalized medical respite program. A limitation of this adjacent literature, however, was oftentimes these studies looked at inner-city postal codes and people experiencing homelessness would rarely be captured in those postal codes [8, 45]. Although this literature did not report any interventions specifically for people experiencing homelessness - a community with its own unique needs and context - this literature did have comparable learnings to our review and insight on how to deliver care to structurally vulnerable groups. Another study by Wong et al. in 2008 reported an elevated risk of CVD in inner-city populations, and they concluded it is not due to a level or lack of awareness of education - in fact, inner city populations could name more CVD risk factors on average than a comparator urban group - and therefore CVD educational and motivational approaches are not sufficient [54]. This links to the tension reported in this review of some providers advocating for more lifestyle interventions for people experiencing homelessness with CVD, while others do not believe the competing priorities will allow for lifestyle changes. Regardless, context-specific data are most beneficial for the homeless population, especially people experiencing homelessness with intersecting structural vulnerabilities such as those who are racialized and homeless.

There were also various articles not included in this review that focused on diabetes management in the homeless community, including learnings on education, empowerment, medication support and supplies, and community engagement [55–57]. Many people experiencing homelessness with CVD also have comorbid diabetes, and it was informative and optimistic that the diabetes literature had a similar approach to management in people experiencing homelessness as CVD. There is a specific pilot program titled “Chronic Disease Prevention and Management Programs [CDM]” in Calgary, Alberta, Canada that uses a partnership-based and community development approach to prevent and manage diabetes in people experiencing homelessness, with the goal of expanding to other chronic diseases that are highly prevalent in the homeless population, including CVD [58]. Perhaps making health care delivery programs that are inclusive of a variety of chronic diseases, to provide comprehensive care in the same visit, is a strong and practical approach.

### Next steps and future research

There are three main calls to action and further research to follow from this review.

First, it was largely highlighted in the literature that the root cause of homelessness is safe, affordable, dignified housing, and that homelessness itself is a risk factor for CVD. We must focus on affordable housing costs, social housing with supports, and government rental assistance [59], to ensure people experiencing homelessness are in stable housing before they can focus on their CVD management and other health priorities.

Second, an emerging theme in the literature was the idea of bringing cardiac specialist care to where the homeless community resides, whether that be in homeless shelter clinics or specialists joining mobile outreach teams. This innovative idea, of bringing cardiology outside of the hospital, must be further researched for feasibility in specific contexts with resource and equipment restraints.

Third, another emerging theme in the literature was digital technology as an avenue for CVD management for people experiencing homelessness, with one reported program using mobile phones for health education and reminders [32]. It is consistent with a study by McInness et al. in 2014 that 89% of homeless veterans surveyed had a mobile phone and 90% of them saying they would be interested in health communication over mobile phone, such as appointment reminders, medication refill reminders, and health checks [60]. A common theme in the literature was that people experiencing homelessness with CVD often need consistent communication with their healthcare team and frequent check-ins, and remote telemonitoring technology for CVD may be suitable for both prevention and management of CVD [5]. This should be an area of further exploration.

### Limitations

This review comes with limitations. First, the usefulness of this scoping review is contingent on research teams and organizations producing a written record of their learnings and programs. It is entirely possible that many organizations have developed and piloted programs and interventions that have led to future recommendations, but the documentation does not exist in the literature or is not available. This highlights the need for more open-access pilot programs so we can learn from one another. Second, the descriptive purpose of this scoping review prevented us from formally assessing risk of bias. Third, while it was of an international scope, most articles were USA-based, and generalizability of these findings needs to be assessed through context-based future research. It is especially relevant when comparing to our own context in Canada with a universal healthcare system. Fourth, we only included articles in English language. Finally, given the qualitative component of our research question, heterogeneity of article type, and the aim not to compare studies but rather accumulate results, we did not do a quality appraisal of articles. We recognize this is a standard of scoping reviews and therefore a limitation.

## Conclusion

The purpose of this review was to gather, synthesize, and disseminate learnings across organizations and countries, so when another group wants to develop a CVD program or approach to care, they can access these learnings. We hope this review is valuable in providing future direction for health care providers, health services research teams, and community organizations. It will certainly inform our future work on CVD management for people experiencing homelessness in Toronto, Canada, with the goal of designing a future program.

There is no ‘one-size fits all’ approach to CVD management for people experiencing homelessness. It is met with complexity, diversity, and intersectionality, including tackling upstream structural factors that contribute to homelessness. However, it is clear we need to move from studying and reporting the CVD burden and inequity in the homeless community, to more practical, community-driven solutions with lived experience at the core.

## Supplementary Information

Supplementary Material 1.

Supplementary Material 2.

## Data Availability

Not applicable.

## Abbreviations

CVD: Cardiovascular disease

## Data availability

No datasets were generated or analysed during the current study.
