# Supplementary material for: Cardiovascular disease management and healthcare delivery for people experiencing homelessness: a scoping review
Source: BMC Health Serv Res. 2024 Sep 17;24:1080. doi: 10.1186/s12913-024-11503-0 (PMC11406789; doi:10.1186/s12913-024-11503-0)
Supplement: Supplementary file 1 — Supplementary Material 1. [file 12913_2024_11503_MOESM1_ESM.docx]

**Additional File 1: Sample Medline Search Strategy**

**Database: Ovid MEDLINE: Epub Ahead of Print, In-Process & Other Non-Indexed Citations, Ovid MEDLINE® Daily and Ovid MEDLINE® <1946-Present>**

--------------------------------------------------------------------------------

1 exp Homeless Persons/ (9223)

2 Emergency Shelter/ (233)

3 homeless*.tw,kf. (11214)

4 fixed address.tw,kf. (38)

5 underhoused.tw,kf. (8)

6 roofless.tw,kf. (15)

7 seeking shelter.tw,kf. (46)

8 unhoused.tw,kf. (19)

9 street involved.tw,kf. (169)

10 sleeping rough.tw,kf. (21)

11 unstabl* hous*.tw,kf. (496)

12 housing instability.tw,kf. (344)

13 precarious* hous*.tw,kf. (76)

14 vulnerably housed.tw,kf. (49)

15 emergency shelter*.tw,kf. (213)

16 housing first.tw,kf. (317)

17 supportive hous*.tw,kf. (401)

18 transitional shelter*.tw,kf. (21)

19 transitional* hous*.tw,kf. (109)

20 marginally housed.tw,kf. (102)

21 skid row.tw,kf. (137)

22 couch surf*.tw,kf. (16)

23 doubled up.tw,kf. (55)

24 street people.tw,kf. (34)

25 living on the street*.tw,kf. (181)

26 or/1-25 (14803)

27 exp Cardiovascular Diseases/ (2421252)

28 exp Cardiology/ (20837)

29 Cardiology Service, Hospital/ (1800)

30 Coronary Angiography/ (66126)

31 exp Cardiovascular Surgical Procedures/ (399877)

32 Cardiac Care Facilities/ (1108)

33 exp Resuscitation/ (96891)

34 exp Diagnostic Techniques, Cardiovascular/ (825330)

35 Cardiovascular Nursing/ (245)

36 Cardiologists/ (731)

37 exp Heart Injuries/ (7077)

38 exp Pacemaker, Artificial/ (27637)

39 exp Cardiovascular System/ (1255409)

40 Heart-Assist Devices/ (14544)

41 Heart Valve Prosthesis/ (36315)

42 exp Cardiovascular Agents/ (1246193)

43 exp Cardiovascular Physiological Phenomena/ (962845)

44 cardiopulmonary bypass/ or heart bypass, left/ (23996)

45 Angioplasty, Balloon, Coronary/ (35503)

46 exp percutaneous coronary intervention/ or exp myocardial revascularization/ (109354)

47 Coronary Care Units/ (4386)

48 exp Coronary Circulation/ (38543)

49 cardio*.tw,kf. (812012)

50 cardiac.tw,kf. (625026)

51 CVD.tw,kf. (38041)

52 coronary.tw,kf. (407864)

53 heart.tw,kf. (861737)

54 myocard.tw,kf. (178)

55 stroke*.tw,kf. (261180)

56 arrhythmia*.tw,kf. (91369)

57 atrial fibrillation.tw,kf. (74286)

58 vascul*.tw,kf. (705080)

59 endocardi*.tw,kf. (51719)

60 pericard*.tw,kf. (43358)

61 ventric*.tw,kf. (419706)

62 aneurysm*.tw,kf. (126588)

63 angio*.tw,kf. (540414)

64 aort*.tw,kf. (296141)

65 arter*.tw,kf. (989936)

66 angina.tw,kf. (54553)

67 isch?em*.tw,kf. (392276)

68 emboli*.tw,kf. (133591)

69 thrombo*.tw,kf. (380837)

70 tachycardi*.tw,kf. (63031)

71 cerebrovascul*.tw,kf. (60082)

72 cerebral vascul*.tw,kf. (8831)

73 resuscitation.tw,kf. (59149)

74 CPR.tw,kf. (12944)

75 hypertension.tw,kf. (401260)

76 hypotension.tw,kf. (55902)

77 ((high* or low* or abnormal) adj1 blood pressure).tw,kf. (33884)

78 (CABG or pacemaker or Embolectom* or Endarterectom* or Endovascular* or Myectomy or Atherectom* or Transmyocardi* or devascular* or microvascular*).tw,kf. (191179)

79 (Valvuloplast* or echocardiograph* or electrocardiograph* or magnetocardiograph* or mechanocardiograph* or phonocardiograph* or radiocardiograph* or capillaroscop*).tw,kf. (203761)

80 or/27-79 (5715404)

81 26 and 80 (553)

82 81 not (Animals/ not (Animals/ and Humans/)) (547)

83 limit 82 to english language (484)

84 remove duplicates from 83 (481)
