# Supplementary material for: Cardiovascular disease management and healthcare delivery for people experiencing homelessness: a scoping review
Source: BMC Health Serv Res. 2024 Sep 17;24:1080. doi: 10.1186/s12913-024-11503-0 (PMC11406789; doi:10.1186/s12913-024-11503-0)
Supplement: Supplementary file 2 — Supplementary Material 2. [file 12913_2024_11503_MOESM2_ESM.docx]

**Additional File 2: Grey Literature Search Strategy**

**Hand-Searched Journals**

Canadian Journal of Cardiology, Heart & Lung, JAMA Network, Canadian Journal of Public Health, NEJM, Journal of Primary Care and Community Health, Journal of General Internal Medicine, Journal of the American College of Cardiology, Circulation, Journal of the American Heart Association, Journal of Health Communication, Journal of Qualitative Research, American Journal of Public Health, Journal of Social Distress and Homelessness, European Journal of Homelessness, International Journal of Equity in Health, Journal of Community Psychology, Journal of Healthcare for the Poor and Underserved, International Journal on Homelessness, BMC Family Practice, American Journal of Managed Care, Quality in Primary Care, Primary Health Care Research & Development, Journal of Family Medicine and Primary Care, Canadian Family Physician, Family Practice, Annals of Family Medicine, BMJ Open, British Journal of General Practice, Journal of American Board of Family Medicine, Social Policy & Society, and Journal of Community Health Nursing.

**Grey Literature Websites**

Homeless Hub, Canadian Observatory on Homelessness, Canadian Alliance to End Homelessness, Canadian Homelessness Research Network, Inner City Health Associates, National Alliance to End Homelessness, National Coalition for the Homeless, National Health Care for the Homeless Council, European Observatory on Homelessness, Homeless Link, Homelessness Australia, Social Interventions Research & Evaluation Network (SIREN), Heart and Stroke Foundation of Canada, Canadian Cardiovascular Society, American College of Cardiology, American Heart Association, European Society of Cardiology, British Heart Foundation, National Heart Foundation of Australia, World Heart Federation.
